# Examining the “Urban Advantage” in Maternal Health Care in Developing Countries

**DOI:** 10.1371/journal.pmed.1000327

**Published:** 2010-09-14

**Authors:** Zoë Matthews, Amos Channon, Sarah Neal, David Osrin, Nyovani Madise, William Stones

**Affiliations:** 1 Division of Social Statistics and Centre for Global Health, Population, Poverty, and Policy, University of Southampton, Southampton, United Kingdom; 2 UCL Centre for International Health and Development, Institute of Child Health, London, United Kingdom; 3 Aga Khan University, Nairobi, Kenya

## Abstract

Andrew Channon and colleagues outline the complexities of urban advantage in maternal health where the urban poor often have worse access to health care than women in rural areas.

**Summary Points**

- Although recent survey data make it possible to examine inequalities in maternal and newborn health care in developing countries, analyses have not tended to take into consideration the special nature of urban poverty.
- Using improved methods to measure urban poverty in 30 countries, we found substantial inequalities in maternal and newborn health, and in access to health care.
- The “urban advantage” is, for some, non-existent. The urban poor do not necessarily have better access to services than the rural poor, despite their proximity to services.
- There are two main patterns of urban inequality in developing countries: (1) massive exclusion, in which most of the population do not have access to services, and (2) urban marginalisation, in which only the poor are excluded. At a country level, these two types of inequality can be further subdivided on the basis of rural access levels.
- Inequity is not mandatory. Patterns of health inequality differ with context, and there are examples of countries with relatively small degrees of urban inequity.
- Women and their babies need to have access to care, especially around the time of birth. Different strategies to achieve universal coverage in urban areas are needed according to urban inequality typology, but the evidence for what works is restricted to a few case studies.

## Introduction

As the global urban population surpasses the rural, continuing growth in most developing countries means an inevitable increase in urban births. The majority of births in many countries will not be in remote rural areas, but in towns and cities [1]. Far from being good news for the twin Millennium Development Goals (MDGs) of maternal and child health—neither of which is currently on track for success [2]—high levels of urbanisation are likely to be associated with increased exclusion from care for many mothers in poor countries, and continued high maternal and newborn mortality among the urban poor. Health and social services in urban areas have not kept pace with urban population growth [3],[4]. Women in slum communities can find care difficult to access even though a well-functioning health infrastructure is located nearby, and in some cases the urban poor have less access to services than people who live in rural areas [5]–[7].

This Policy Forum article investigates the “urban advantage” to determine whether the urban poor in a range of different countries really do have an advantage over rural populations in health and access to services. It also quantifies the gap between the urban poor and other residents of towns and cities. We investigate whether the urban rich–poor gap is a feature of all cities, or whether there are some countries whose urban environments are more equitable than those of others.

Using nationally representative Demographic and Health Survey data from 30 developing countries in Africa, Asia and Latin America (representing approximately 47% of developing country populations), we look at maternal and newborn service use among groups with different levels of poverty. Most previous studies have not distinguished between poverty groups in urban settings and those in rural areas, categorising the poor as mainly rural. Our study takes into account the different nature of deprivation in urban areas, and identifies health access indicators among the urban poor separately from the equivalent indicators for rural populations. It also shows patterns of inequalities within cities in different countries, and explores the ways that urban and rural inequalities interlink. By identifying patterns of unequal access to services for mothers and babies, we pinpoint barriers to access in these different contexts, and conclude by suggesting evidence-based policy solutions where documented.

## Is There an Urban Advantage in Maternal and Newborn Health, and How Large Is the Gap between Rich and Poor in Cities?

Over the last few decades, large-scale migration from rural to urban areas in developing countries has led to a proliferation of slums and informal settlements in many cities and towns. High fertility in urban areas, especially in poorer groups, has further boosted city populations. Cities are not only becoming larger, they are becoming more inequitable, with large impoverished and marginalised settlements springing up often in close proximity to relatively wealthy existing communities. Much of the existing literature has tended to ignore these inequities and focus instead on simple average differences between urban and rural areas, indicating that most nations experience substantially better maternal and neonatal survival in urban than in rural areas [8]–[11]. The urban–rural difference is often explained by the greater access to health care services available to urban residents, and this is indeed supported by a number of studies [8],[12]–[15].

However, recent studies have suggested that urban populations are changing in many countries, leading to the possibility of an erosion of the urban health advantage and increasing concerns about the re-emergence of an “urban penalty” that was assumed to have been consigned to history [16]. Poor and marginalised urban subgroups compare unfavourably with other urban dwellers with respect to mortality [17]–[20], and groups such as the poorest migrants from rural areas and slum dwellers may have maternal, newborn and child mortality rates as high as or even higher than the rural poor [4],[21]–[24].

Few studies have looked at inequalities within urban areas, or quantified urban poverty adequately, although it is possible to do so using survey data. Wealth can be difficult to capture, but households can be classified into five equal groups (quintiles) according to their asset wealth [25] where information on household expenditure, income, or consumption is not available [26]. Asset wealth is usually calculated across whole populations without accounting for location. However, asset wealth differs considerably between urban and rural areas, with agricultural, livestock and land assets more important in rural areas than in cities. Using recent survey data, however, it is possible to derive asset quintiles for urban and rural areas separately, and thus provide a better way to identify poor populations in both settings. Separate asset quintiles have been calculated throughout this paper to quantify inequalities.

**Figure 1:**
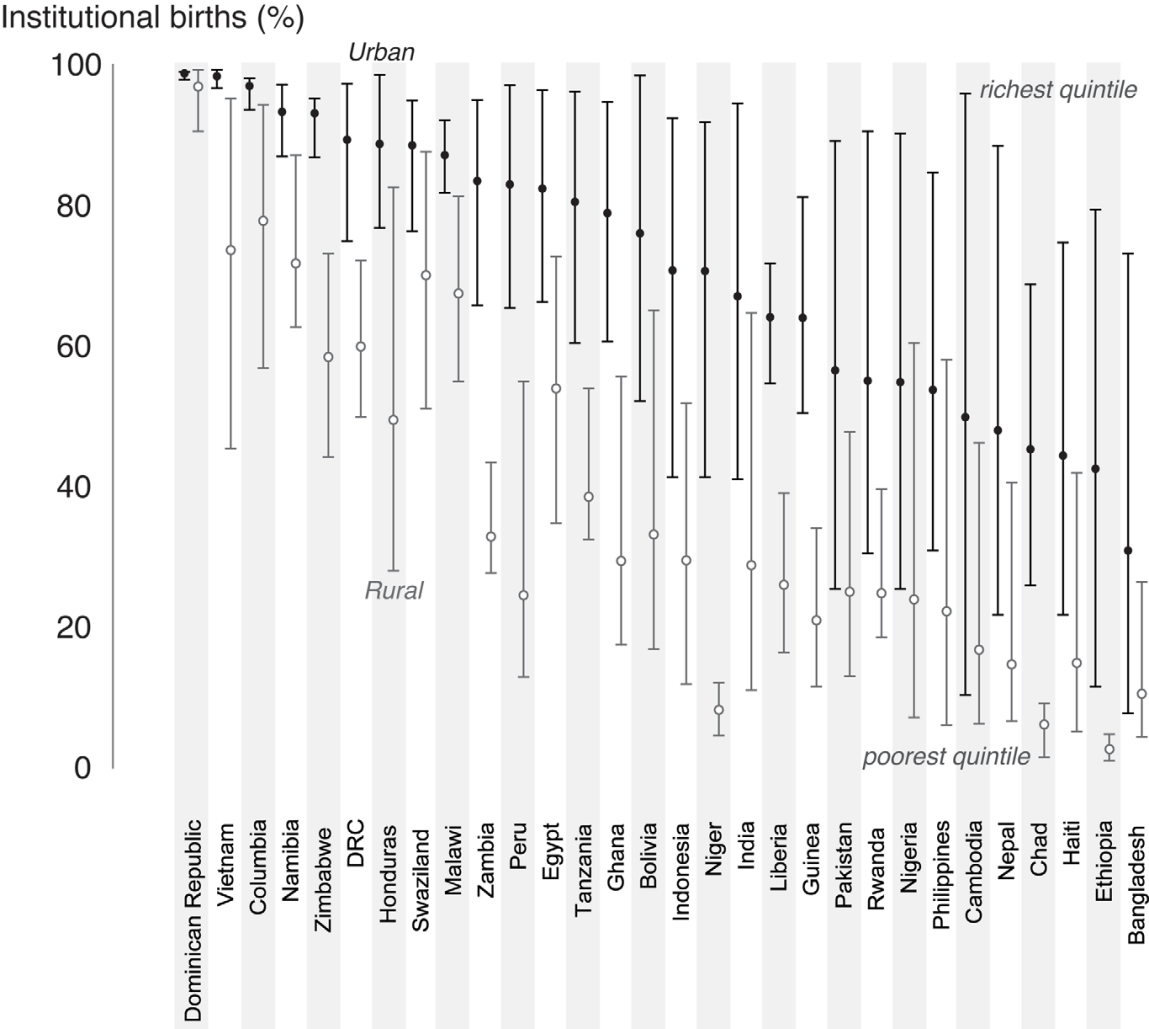
Percentage of births in facilities by place of residence in 30 countries showing the highest and lowest wealth quintiles.

The proportion of women that give birth in a health facility, a key measure of service coverage for both mothers and newborns, can be calculated for each quintile to investigate the nature of the urban advantage and the magnitude of urban inequality. Figure 1 shows this indicator for the poorest and the richest asset quintiles in 30 low-income countries. It allows comparison between countries, between urban and rural areas, and between the richest and poorest groups in each country.

The poor–rich gap, although often wide in rural areas, is sometimes much wider in urban areas. In some countries, such as Cambodia and Nepal, a facility birth rate of over 80% for the urban rich coexists with rates of around 20% for women in the poorest quintile. Comparing urban inequalities with those in rural areas, we see two distinct tendencies. Firstly, there are countries where urban living confers a distinct advantage, leaving behind a rural situation where most are excluded from care (for example, in Ethiopia, Chad and Niger). In contrast, there are other countries where rural inequalities are large, and there is a large overlap between urban and rural areas, suggesting that the urban poor are being left behind as the richest of rural households catch up.

## Are There Identifiable Patterns of Urban Exclusion for Which Different Policies and Strategies Could Be Developed?

**Figure 2:**
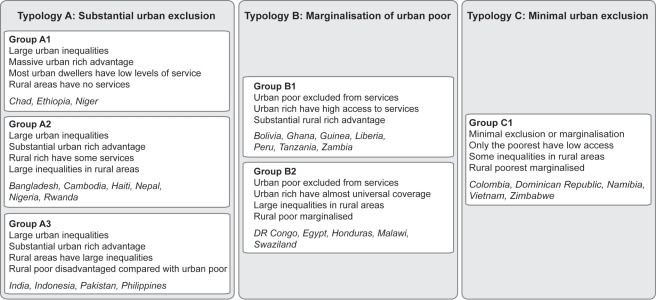
Typologies for urban coverage of maternal-newborn services.

**Figure 3:**
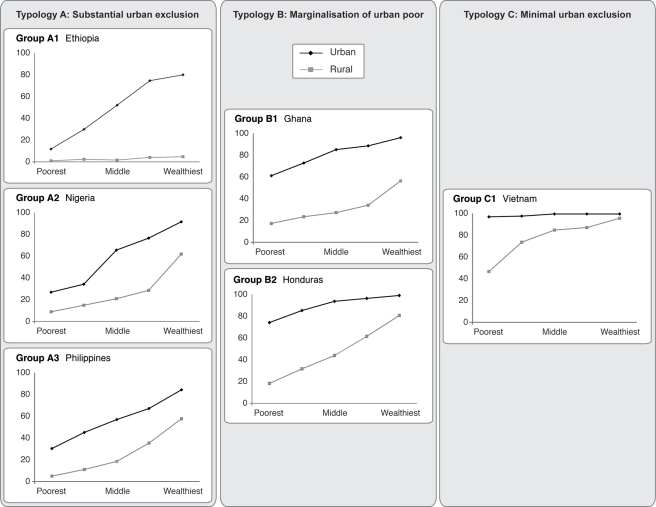
Percentage of births in facilities by wealth quintile in urban and rural populations; examples of countries in each typology.

Several patterns or typologies of exclusion from care emerge from the data on health service use. These are characterised not only by inequalities in urban areas, but also by the variability of health service access in the rural areas that feed them. Three different urban scenarios can be distinguished, and are summarised in Figure 2. First, there are countries with a very large exclusion problem, where it is not only the poor who are excluded, but many others as well. These are countries with urban areas where less than 75% of mothers give birth in a health facility. Many countries fall into this “substantial urban exclusion” category, although they show a spectrum of concurrent rural service use: some with almost non-existent rural services, others where the rural rich have more access to services than the urban poor. The second scenario is where there is marginalisation of the urban poor. In these countries a high proportion of urban residents obtain health services, but most of the very poorest group do not. In the third group of countries, the urban population is well served across the socioeconomic spectrum with little inequality, representing a situation moving rapidly towards the gold standard of universal health provision for mothers and babies. Figure 3 shows example countries in each of these three scenarios to illustrate the very different nature of inequality that exists in developing countries.

**Figure 4:**
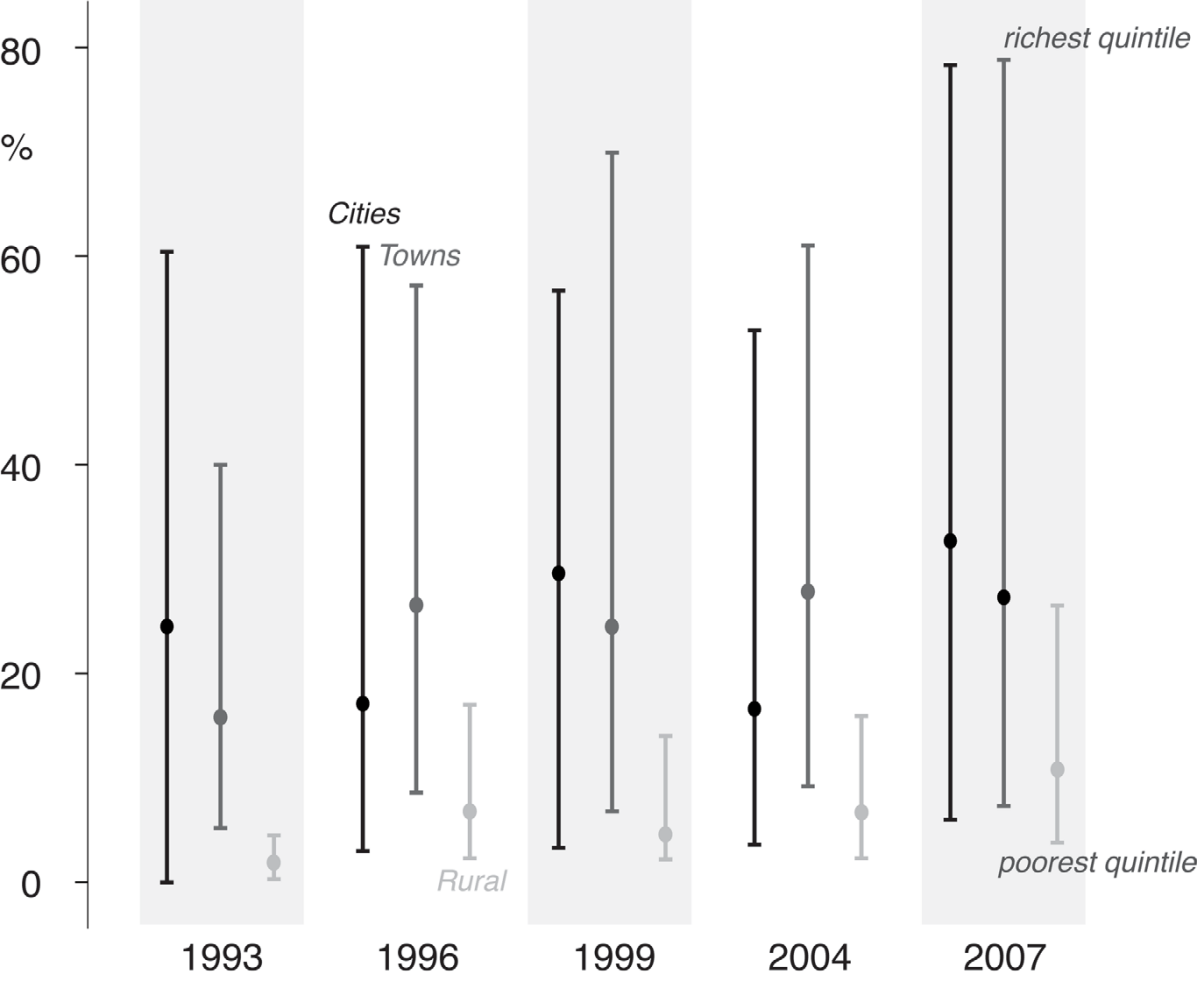
Percentage of facility births by place of residence over time in Bangladesh showing the highest and lowest wealth quintiles.

Different policy solutions are necessary according to the inequality pattern that prevails. Some of these patterns are tied to a geographical region (Box 1). For example, exclusion patterns for the poor in urban Asia tend to have different characteristics from those seen in Latin America. Also, the patterns of exclusion observed are clearly not static, with most countries moving towards universal coverage in both urban and rural areas, albeit at very different speeds. Hence, with the right policies, a country could move from group A1, with substantial urban exclusion, to group C1 with minimal urban exclusion, and beyond to universal coverage. Change can, however, take some time, and inequalities are not necessarily reduced along the way. For example, Bangladesh has moved over a 14-year period from a position of massive urban inequality and minimal rural service use in 1993 (Group A1) to one in which urban inequality has increased. By 2007, the rural rich had also started to benefit from increased service access, so that the 14 years since the start of the MDG timeframe have only resulted in a transition from Group A1 to A2, with hardly any progress in tackling the growing problem of the urban poor in cities with many slum settlements such as Dhaka (Figure 4). Clearly, urban areas are not homogeneous entities. They include capital cities, large and smaller cities, and towns, and some surveys do include data on types of urban settings. Bangladesh is one of these, and Figure 4 shows that the urban inequalities remain regardless of the size of the city or town. Put simply, few urban environments escape the exclusion that is now part of the lives of millions of slum dwellers and the poor worldwide.

**Box 1. Key Policy Recommendations**

1. The numbers of urban poor are increasing, and improved access to basic health care services is needed to reduce large and increasing inequalities in urban areas, and to ensure that women and children have access to care, especially around the time of birth.
2. Inequalities between the urban rich and poor do not have to exist. In some poor countries there is little difference between urban communities in access to care. These countries have rolled out universal access to care and, given political will, this can be done in countries with low GDP.
3. Strategies to address the known barriers to service use—geographical distance, cost, lack of services and poor staffing—should be tailored to the inequality context. In situations of massive urban deprivation, the health system needs to be expanded beyond the reach of only the rich, and in situations of marginal exclusion of the poor, targeted services and financial protection schemes are needed. Insurance or voucher schemes have had some success in opening up access to marginalised populations.
4. Urban inequalities should be examined alongside corresponding rural inequalities due to the inter-relationship between the two areas and as a matter of social equity.
5. Improvement of service quality should be supported by regulation and standards in both public and private sectors.

## Reasons for Inequalities and Documented Barriers to Care for the Urban Poor

The choice of strategies to improve coverage of care in the towns and cities of developing countries depends on the pattern of exclusion. Understanding the barriers to care for the urban poor is the first step towards building strategies, although only limited evidence exists to guide policymakers. Countries where substantial exclusion from maternal and newborn care is seen in urban environments are generally suffering from an inadequate urban health infrastructure. Although there is evidence of cultural barriers constraining care-seeking [27],[28], as well as gender factors [29] and lack of knowledge on the part of women and their families [30], it is clear that where enough care has been provided to the majority of women it is almost universally accessed in urban areas, even by recent migrants.

For those settings where there is a marked marginalisation rather than wholesale exclusion, barriers to care are somewhat different. Geographical constraints might affect certain localities and marginal groups, even within urban areas [5],[19],[28]. Financial access can be even more important for the poor [18],[28],[31]. Gupta et al. noted that while slum dwellers in Chandigarh had geographical access to quality public facilities, their access was restricted by the need to pay for prescriptions [5]. A study by Hossain and Hoque found that 26% of women in urban slums in Dhaka cited high informal costs as reasons why they did not make use of the ostensibly free hospital delivery services, and that even marginal differences in economic well-being had an important impact on use of delivery care [27].

Apart from geographical and financial constraints, marginalisation can be more simply understood as pure discrimination against the poor. Studies suggest that the poor in urban environments may receive disproportionately lower standards of care even when they use the same facilities as their more wealthy neighbours, with the ability to pay affecting both the quality and timeliness of care even in government hospitals [32]. Poor treatment translates into reluctance to use services, and ultimately the poor are marginalised when they perceive care to be rude, neglectful, indifferent, or even abusive [9],[27],[33].

One characteristic of urbanised health care is the proliferation of private health facilities, and, particularly in the absence of closely located public services, a number of studies show that poor urban women are more likely to use private than public services [20],[34]. As more qualified practitioners may be expensive, the poor are often forced to use cheaper, less qualified, and often unregulated providers [35],[36], opening up inequalities in survival and effectively marginalising substantial subgroups from care that they cannot afford.

Even where services are abundant and financially accessible, they may not be particularly popular, for the simple reason that they are often neither staffed nor stocked adequately. Substantial exclusion can occur where infrastructure is particularly weak, or the disproportionate effect of inadequate services on the poor can result in their marginalisation. Fotso et al. found 17 private facilities located within a Nairobi slum, none of which could provide even basic emergency obstetric care. While 70% of women gave birth at health facilities, only 48% did so at facilities with minimum standards of care and provision of basic emergency obstetric care [37]. Studies have indicated shortcomings in both public and private health services in poor areas of cities, including shortages of drugs, lack of facilities to support surgery (such as blood banks), health workers not present during births, and inappropriate procedures, exacerbated by lower levels of competence [33],[36],[38].

## Are There Examples of What Works in Different Contexts to Eliminate Urban Inequality for Mothers and Newborns?

Understanding the barriers to access for women and their babies is the first step, but there is little evidence to guide interventions aimed at breaking them down in urban areas. Strategies for breaking through the constraints should be tailored to context and exclusion typology. Setting up outreach or insurance systems for a minority group of poor and marginalised families is a very different proposition from the establishment of services over a wide range of urban population groups. According to the concurrent situation in rural areas, there may be migration streams with different expectations and demands in terms of service availability. Expanding services for the poor who are recent migrants from rural areas where services are totally lacking requires a different approach from service provision for rural migrants who have already attained a level of access in their communities of origin.

In contexts where substantial proportions of the urban population are excluded from care, the solution is mainly to build and expand services. Indeed, rolling out urban services can be considered easier than rural expansion—given proximity to resources—but training competent staff and ensuring effective infrastructure, supplies and commodities are not straightforward issues even in urban areas. In many settings the public sector is inadequate, with perverse incentives in crowded facilities whereby overloaded staff may “deter” users, through rudeness or poor communication. The interaction between health care provider and client goes beyond the purely clinical, and poor motivation and communication are a significant disincentive to consultation: this is at least part of the reason for the drift to the private sector. Improvement in interpersonal quality of care needs to be a key component of system strengthening [39]. There needs to be a closer match between volumes and staffing levels, especially on the “front line” of birthing services, so that providers have an incentive to draw in and welcome clients. Unregulated private services in urban areas do not currently fill this gap, and a lack of competent staff in urban facilities exacerbates the problem of poor client–provider communication.

Part of the solution to expanding urban care in scenarios of massive exclusion is to tackle the human resource crisis, in terms of both providing more workers and improving the skill mix. However, whilst moves to promote task shifting among providers by empowering nurses and clinical officers to undertake duties previously performed by doctors can be very effective, they can also be dangerous if unregulated [40]. In a context of restricted health care expenditure in the public sector, the urban setting in many countries is characterised by poorly remunerated or very limited employment of graduating doctors, nurses and clinical officers who could fill the service gaps. At present, many of these unemployed or poorly paid health professionals have to seek a livelihood in the unregulated private sector or find opportunities for migration in order to make ends meet. A first step is to recognise the magnitude of the unmet need for services in these settings where the urban poor are substantially excluded, and invest in both the quality and quantity of service provision, free at the point of use, that has flexibility to expand without loss of quality as utilisation increases. Decentralisation and health care reforms in many settings can be an opportunity for authorities in large and small cities to become more attuned to the particular characteristics of their urban setting. However, inexperienced municipalities may be taking over local health care planning, and may contract care out to poor quality private providers who are not able to serve the poor.

It must be acknowledged that private health provision will continue to expand in many countries, and it is important to engage with these providers to maximise the possible opportunities for improving access to quality health care for the urban poor. For instance, the World Bank Health in Africa Initiative is developing a strategy to expand socially responsible private provision through a number of measures, including financial investment in appropriate health care companies serving low-income groups, supporting improved mechanisms for regulation, and working with governments to develop public–private partnerships [41].

When drawing up strategies to tackle substantial exclusion, the situation in rural areas of the same country may need to be taken into account. Recent data show that massive urban exclusion can be accompanied by an almost total lack of rural service use in some countries. For these settings rolling out care and ensuring financial access is a priority in both urban and rural areas.

Countries with marginalisation of urban poor populations are characterised by better available infrastructure and service capacity, but also by the presence of specific marginalised groups that have limited access to the available services. Promising approaches to reach marginalised groups include voucher schemes for free care at the point of service to a defined standard of quality. Initiatives in Kenya have suggested this approach has scope for improving access for the poorest, but further evaluation is needed. Other innovative ways of reaching the underserved have been suggested but not widely tested, especially in urban contexts [42].

Urban environments are characterised by ready commodity markets for those who can afford them. Food, water, electricity, education, health care and time are competing demands for the limited cash flow of slum dwellers. In some cases, the availability and quality of health care for mothers and newborn infants is limited across the board. In others, the deficiencies affect poor and marginalised families disproportionately. In almost all developing countries, the urban public sector has to cope with a rising tide of births to the poorer and more vulnerable. This affects the case mix and the perceptions of clients, who vote with their feet by using the unregulated private sector when finances allow. Municipalities and governments face two major challenges: firstly, closing the exclusion gap, which implies significant expansion of services in those contexts where large proportions of the population do not access care, and within a context of marginalisation there is a need for targeted approaches to improve the quality and uptake of care by the neediest. Secondly, there is a critical challenge to universalize quality with corresponding benchmarking and regulation in both sectors.

## References

[1] United Nations Department of Economic and Social Affairs Population Division (2009). World population prospects: the 2008 revision population database..

[2] United Nations (2009). The Millennium Development Goals report..

[3] Montgomery MR (2009). Urban poverty and health in developing countries..

[4] Fotso JC, Ezeh A, Madise N, Ciera J (2007). Progress towards the child mortality millennium development goal in urban sub-Saharan Africa: the dynamics of population growth, immunization, and access to clean water.. BMC Public Health.

[5] Gupta M, Thakur J, Kumar R (2008). Reproductive and child health inequities in Chandigarh Union Territory of India.. J Urban Health.

[6] African Population and Health Research Centre (APHRC) (2002). Health and livelihood needs of residents of informal settlements in Nairobi City..

[7] UN-HABITAT (2006). State of the world's cities 2006/7..

[8] Ministry of Health and Population (2001). The national maternal mortality study Egypt, 2000..

[9] Bartlett LA, Mawji S, Whitehead S, Crouse C, Dalil S (2005). Where giving birth is a forecast of death: maternal mortality in four districts of Afghanistan, 1999–2002.. Lancet.

[10] Ronsmans C, Graham W (2006). Maternal mortality: who, when, where and why?. Lancet.

[11] Mahy M (2003). Childhood mortality in the developing world: a review of evidence from the Demographic and Health Surveys..

[12] Ronsmans C, Etard JF, Walraven G, Høj L, Dumont A (2003). Maternal mortality and access to obstetric services in West Africa.. Trop Med Int Health.

[13] Say L, Raine R (2007). A systematic review of inequalities in the use of maternal health care in developing countries: examining the scale of the problem and the importance of context.. Bull World Health Organ.

[14] Thaddeus S, Maine D (1994). Too far to walk: maternal mortality in context.. Soc Sci Med.

[15] Central Bureau of Statistics (CBS) [Kenya], Ministry of Health (MOH), ORC Macro (2004). Kenya demographic and health survey 2003..

[16] Harpham T (2009). Urban health in developing countries: what do we know and where do we go?. Health Place.

[17] Hu X, Cook S, Salazar M (2008). Internal migration and health in China.. Lancet.

[18] Zhao Q, Kulane A, Gao Y, Xu B (2009). Knowledge and attitude on maternal health care among rural-to-urban migrant women in Shanghai, China.. BMC Womens Health.

[19] Ziraba A, Madise N, Mills S, Kyobutungi C, Ezeh A (2009). Maternal mortality in the informal settlements of Nairobi city: what do we know?. Reprod Health.

[20] Shah More N, Bapat U, Das S, Barnett S, Costello A (2009). Inequalities in maternity care and newborn outcomes: one-year surveillance of births in vulnerable slum communities in Mumbai.. Int J Equity Health.

[21] Madise N, Banda EM, Benaya KW (2003). Infant mortality in Zambia: socio-economic and demographic determinants.. Soc Biol.

[22] Madise N, Diamond I (1995). Determinants of infant mortality in Malawi: an analysis to control for death clustering within families.. J Biosoc Sci.

[23] Van de Poel E, O'Donnell O, Van Doorslaer E (2007). Are urban children really healthier? Evidence from 47 developing countries.. Soc Sci Med.

[24] Urban Health Resource Centre (2006). State of urban health in Delhi..

[25] Filmer D, Pritchett L (1998). Estimating wealth effects without expenditure data - or tears: With an application to educational enrollment in states of India..

[26] Falkingham J, Namazie C (2002). Measuring health and poverty: a review of approaches to identifying the poor..

[27] Hossain I, Hoque M (2005). Determinants of choice of delivery care in some urban slums of Dhaka City.. Pakistan Journal of Social Sciences.

[28] Agarwal S, Bhanot A, Sangar K (2005). Neonatal care and transport among the urban poor: Challenges and options.. Journal of Neonatology.

[29] Matthews Z, Brookes M, Stones RW, Hossain MB, Moore S (2005). Village in the city: autonomy and maternal health-seeking among slum populations of Mumbai.. A focus on gender: collected papers on gender.

[30] Bajaj J (1999). Knowledge and utilisation of maternal and child health services in Delhi slums.. Journal of Family Welfare.

[31] Khan Z, Mehnaz S, Khalique N, Ansari M, Siddiqui A (2009). Poor perinatal care practices in urban slums: possible role of social mobilization networks.. Indian Journal of Community Medicine.

[32] Pitchforth E, van Teijlingen E, Graham W, Dixon-Woods M, Chowdhury M (2006). Getting women to hospital is not enough: a qualitative study of access to emergency obstetric care in Bangladesh.. Qual Saf Health Care.

[33] Hulton L, Matthews Z, Stones RW (2007). Applying a framework for assessing the quality of maternal health services in urban India.. Soc Sci Med.

[34] Shah More N, Alcock G, Bapat U, Das S, Joshi W (2009). Tracing pathways from antenatal to delivery care for women in Mumbai, India: cross-sectional study of maternity in low-income areas.. Int Health.

[35] Fernandez A, Mondkar J, Mathai S (2003). Urban slum-specific issues in neonatal survival.. Indian Pediatr.

[36] Das J, Hammer J (2007). Money for nothing: the dire straits of medical practice in Delhi, India.. Journal of Development Economics.

[37] Fotso JC, Ezeh A, Oronje R (2008). Provision and use of maternal health services among urban poor women in Kenya: what do we know and what can we do?. J Urban Health.

[38] Das J, Hammer J (2007). Location, location, location: residence, wealth and the quality of medical care in Delhi, India.. Health Aff (Millwood).

[39] Fernandez A, Osrin D (2006). The city initiative for newborn health.. PLoS Med.

[40] FIGO Safe Motherhood and Newborn Health Committee (2009). Human resources for health in the low-resource world: collaborative practice and task shifting in maternal and neonatal care.. Int J Gynaecol Obstet.

[41] International Finance Corporation / World Bank Group (2009). http://www.ifc.org/ifcext/media.nsf/AttachmentsByTitle/AM09_AfricaHealthCare/FILE/AM09_AfricaHealthCare.pdf.

[42] Griffiths D, Bryant L, Arkuta S (2009). Innovative reproductive health service delivery: models for underserved communities. International Journal of Gynecology and Obstetrics 107, Supplement 2: S34 Abstract No. I139.

